# RepA Promotes the Nucleolar Exclusion of the V2 Protein of Mulberry Mosaic Dwarf-Associated Virus

**DOI:** 10.3389/fmicb.2020.01828

**Published:** 2020-08-04

**Authors:** Dongxue Wang, Shaoshuang Sun, Yanxiang Ren, Shifang Li, Xiuling Yang, Xueping Zhou

**Affiliations:** ^1^State Key Laboratory for Biology of Plant Diseases and Insect Pests, Institute of Plant Protection, Chinese Academy of Agricultural Sciences, Beijing, China; ^2^State Key Laboratory of Agro-Biotechnology and Ministry of Agriculture Key Laboratory of Soil Microbiology, College of Biological Sciences, China Agricultural University, Beijing, China; ^3^State Key Laboratory of Rice Biology, Institute of Biotechnology, Zhejiang University, Hangzhou, China

**Keywords:** geminivirus, MMDaV, V2, RepA, nucleolus, protein–protein interactions

## Abstract

Plant viruses have limited coding capacities so that they rely heavily on the expression of multifunctional viral proteins to achieve a successful infection. The functional specification of viral proteins is often related to their differential interaction with plant and viral components and somewhat depends on their localization to various subcellular compartments. In this study, we analyzed the intracellular localization of the V2 protein of *Mulberry mosaic dwarf-associated virus* (MMDaV), an unsigned species of the family *Geminiviridae*. We show that the V2 protein colocalizes with the nucleolar protein fibrillarin (NbFib2) in the nucleolus upon transient expression in the epidermal cells of *Nicotiana benthamiana*. A yeast-two hybrid assay, followed by bimolecular fluorescence complementation assays, demonstrated the specific interaction between V2 and NbFib2. Intriguingly, we find that the presence of MMDaV excludes the V2 protein from the nucleolus to nucleoplasm. We present evidence that the replication-associated protein A (RepA) protein of MMDaV interacts with V2 and enables the nucleolar exclusion of V2. We also show that, while V2 interacts with itself primarily in the nucleolus, the presence of RepA redirects the site of V2–V2 interaction from the nucleolus to the nucleoplasm. We further reveal that RepA promotes V2 out of the nucleolus presumably by directing the NbFib2-V2 complex from the nucleolus to the nucleoplasm. Considering the critical role of the nucleolus in plant virus infection, this RepA-dependent modulation of V2 nucleolar localization would be crucial for understanding the involvement of this subcellular compartment in plant–virus interactions.

## Introduction

Plant viruses are obligate intracellular parasites that have long caused significant yield losses to crops and are continuously threatening crop cultivation worldwide. With limited coding capacities, plant viruses usually encode multifunctional viral proteins that play important roles in several steps of a virus life cycle. The functional specification of these multifunctional viral proteins is often related to their differential interaction with host plant and viral components and somewhat depends on their dynamic localization to various subcellular compartments (Medina-Puche and Lozano-Duran, 2019). Understanding how viral proteins manipulate plant subcellular organelles would provide cues to depict protein function *in vivo*.

The plant nucleolus is the prominent subnuclear structure of cells that comprises several functional subcompartments such as fibrillar centers, a dense fibrillary component, a granular component, nucleolar vacuoles, nucleonema, and nucleolar chromatin (Stepinski, 2014). Besides its well-known role in ribosome RNA synthesis, the nucleolus plays critical roles in cell cycle regulation and responses to biotic and abiotic stresses (Kalinina et al., 2018). Although many plant RNA viruses replicate in the cytoplasm of host cells, an increasing number of viral proteins have been shown to localize, at least at some stage during infection, in the nucleolus (Hiscox, 2007). Examples include the umbravirus groundnut rosette virus (GRV) ORF3 protein (Kim et al., 2007), barley stripe mosaic virus (BSMV, hordeivirus) Triple Gene Block1 (TGB1) (Li et al., 2018), potyvirus VPg (Rajamäki and Valkonen, 2009), rice stripe virus (tenuivirus) P2 (Zheng et al., 2015), and the P20 protein encoded by bamboo mosaic virus (BaMV, potexvirus) satellite RNA (satRNA) (Chang et al., 2016). Viral proteins may subsequently utilize nucleolar functions for assembly of viral ribonucleoprotein (RNP) particles, virus replication, and movement, and to counteract plant antiviral defense (Kalinina et al., 2018).

Unlike RNA viruses, geminiviruses are a group of circular single-stranded DNA (ssDNA) viruses that replicate in the nucleus. Based on their genome organization, host range, and insect vector, geminiviruses have been classified into nine genera, namely, *Becurtovirus*, *Begomovirus*, *Capulavirus*, *Curtovirus*, *Eragrovirus*, *Grablovirus*, *Mastrevirus*, *Topocuvirus*, and *Turncurtovirus* (Zerbini et al., 2017). The genome of geminiviruses contains either one or two DNA molecules (2.7–3.0 kb) that is (are) encapsidated in twinned icosahedral particles. After uncoating, viral ssDNA is released into host cells and subsequently converted into double-stranded DNA (dsDNA) in the nuclei of differentiated plant cells (Gutierrez, 1999; Jeske, 2009). These dsDNAs can be packaged into viral minichromosomes that serve as templates for transcription, further replication, and the synthesis of progeny ssDNA that is encapsidated by the capsid protein to form virions (Gutierrez, 1999; Jeske, 2009; Hanley-Bowdoin et al., 2013). Assembly of geminivirus particles also occurs in the nucleus, and large aggregates consisting of geminivirus particles have been observed in the nuclei of infected plant cells (Rushing et al., 1987). Due to the extensive reliance of geminivirus life cycle on nuclear activities, many geminivirus proteins, such as Replication initiator protein (Rep) and transcription activator protein, target to the plant nucleus (Maio et al., 2019). As the nucleolus is the critical sub-domain of a plant nucleus, it is reasonable that the nucleus-replicating geminiviruses could interact with the immediately accessible nucleolus. For example, coat protein (CP), the unique protein required for geminivirus capsids formation, insect vector transmission, and nuclear shuttling of viral DNA of monopartite geminiviruses, is accumulated mainly in the nucleolus of both insect and tobacco cells (Kunik et al., 1998; Fondong, 2013). Reminiscent of the differential localization of CPs associated to the infection of animal DNA viruses, such as ssDNA Adeno-associated virus type 2 (Wistuba et al., 1997) and dsDNA human polyomavirus JC (Shishido-Hara et al., 2000), CP of a begomovirus tomato yellow leaf curl virus (TYLCV) is temporally excluded outside of the nucleolus in the progression of virus infection (Wang et al., 2017). This dynamic virus-dependent subnuclear localization of CP might underpin its different functions, highlighting the importance of observing the subcellular localization of viral proteins in the context of virus infection.

The V2/AV2 open reading frame, located upstream of the *CP* gene, is present in the virion-sense strand of different geminiviruses but not in members of the New World bipartite begomoviruses. The V2/AV2 protein of several geminiviruses has been identified as a suppressor of posttranscriptional gene silencing (PTGS) and transcriptional gene silencing (TGS) (Glick et al., 2008; Wang et al., 2014, 2018, 2019; Luna and Lozano-Durán, 2020). V2/AV2 is also involved in viral systemic infection, possibly by interacting with CP and facilitating nuclear export of CP to shuttle CP-mediated DNA export between the nucleus and cytoplasm (Poornima et al., 2011; Zhao et al., 2020). Although some functions of V2/AV2, such as the role in suppression of PTGS, are conserved across tested geminivirus species and genera (Luna and Lozano-Durán, 2020), the subcellular localization of V2/AV2 seems to vary in different species. The GFP-tagged V2 protein of monopartite begomoviruses, TYLCV, tomato leaf curl virus, tomato leaf curl Java virus, and a curtovirus beet curly top virus, is reported to localize to the perinuclear, cytoplasm, and endoplasmic reticulum of plant cells (Rojas et al., 2001; Sharma et al., 2011; Luna et al., 2017). TYLCV V2 is also demonstrated to form cytoskeleton-dependent aggregates that play an important role in TYLCV infection (Moshe et al., 2015), and to interact with AGO4 in the cajal body to suppress TGS (Ding and Lozano-Duran, 2020; Wang et al., 2020). The AV2 protein of a bipartite begomovirus, Indian cassava mosaic virus (ICMV), targets to cell periphery and to punctate spots that indicate plasmodesmal localization. ICMV AV2 also accumulates in nuclei in pairs of globular inclusions before it reaches the cells’ periphery and the adjacent cells (Rothenstein et al., 2007). Grapevine red blotch-associated virus (GRBaV) V2 localizes to the nucleoplasm, Cajal bodies, and cytoplasm, and is redirected to the nucleolus upon coexpression with the nucleus and Cajal body-associated protein fibrillarin 2 (Fib2) (Guo et al., 2015). The V2 protein of a distinct monopartite geminivirus, Mulberry mosaic dwarf-associated virus (MMDaV), was shown to localize to both subnuclear foci and cytoplasm of the epidermal cells of *Nicotiana benthamiana* plants (Yang et al., 2018). Most of these experiments, however, have studied the subcellular localization of V2 in the absence of virus infection. Whether the localization of V2 is different in the context of virus infection remains to be evaluated. Since MMDaV V2 shows low sequence homology with the V2 proteins encoded by other geminiviruses (Ma et al., 2015), it is particularly interesting to investigate its subcellular localization in detail.

In this study, we analyzed the dynamic subcellular localization of MMDaV V2 in *N. benthamiana* in the absence or presence of MMDaV infection. We show that the MMDaV V2 protein colocalizes with the nucleolar protein fibrillarin in the nucleolus upon transient expression of V2 in the epidermal cells of *N. benthamiana*. Strikingly, we find that the V2 protein is excluded from the nucleolus to nucleoplasm in the context of MMDaV infection. We also demonstrate that the RepA interacts with V2 and mediates the change of V2 subcellular localization.

## Materials and Methods

### Plant Growth

Wild-type *N. benthamiana* and transgenic *N. benthamiana* plants expressing a red fluorescent protein (RFP)-tagged histone 2B nuclear marker protein (RFP-H2B plants) (Martin et al., 2009) were grown in an insect-free growth room at 25°C under a 16 h light/8 h dark cycle.

### Plasmid Construction

The *RepA*, *V2*, and *V3* genes from MMDaV (GenBank accession number KP303687) (Ma et al., 2015), the V2^dm61–77aa^ from pGEM-T-V2^dm61–77aa^ (Yang et al., 2018) and the coding sequences of the *fibrillarin 2* gene of *N. benthamiana* cDNA (GenBank accession number AM269909, NbFib2) were cloned into pENTR-D/TOPO entry vector without stop codon by standard protocols (Invitrogen, Beijing, China) to yield pENTR-RepA, pENTR-V2, pENTR-V3, pENTR-V2^dm61–77aa^, and pENTR-NbFib2, respectively. For subcellular localization analysis, NbFib2 and RepA was gateway-cloned into plant binary vectors pEarleyGate102 [N-terminal cyan fluorescent protein (CFP) fusion] and pGWB454 (N-terminal RFP fusion) to yield Fib2-RFP and RepA-CFP, respectively (Earley et al., 2006; Nakagawa et al., 2007). The expression vectors, GFP-V2 and GFP-V2^dm61–77aa^, containing fusions to an enhanced green fluorescent protein (eGFP) were constructed previously (Yang et al., 2018). To produce bimolecular fluorescence complementation (BiFC) vectors, the entry clones were transferred to gateway compatible 201-YN vector to yield a fusion with the N-terminal fragment of yellow fluorescent protein (nYFP) or the 201-YC vector as a fusion with the C-terminal fragment of YFP (cYFP) as described (Lu et al., 2010). To generate the bait and prey plasmids for yeast two-hybrid assays, the full-length fragment of RepA, V2, and NbFib2 was amplified using TransStart FastPfu high-fidelity DNA polymerase following the manufacturer’s instructions (Transgene, Beijing, China) and then cloned into the *Eco*RI-*Bam*HI site of the yeast GAL4 DNA-binding domain vector pGBKT7 and the yeast GAL4 activation domain vector pGADT7 (Clontech, China), respectively. The oligonucleotide primers used to generate the plasmids are listed in Supplementary Table S1. All inserts were confirmed by DNA sequencing.

A two-tandem repeat of MMDaV was cloned into the pBinPLUS vector to generate the infectious clone of MMDaV (Yang et al., unpublished). The recombinant plant binary pCHF3 vectors expressing the individual ORFs of MMDaV (*V1, V3, V4, V5, RepA*, and *Rep*) were constructed in a previous study (Yang et al., 2018).

### Transient Expression by Agro-Infiltration

The resulting plant expression constructs were individually transformed into the *Agrobacterium tumefaciens* strain C58C1. The infectious clone of MMDaV was electroporated into *A. tumefaciens* strain EHA105. Agrobacteria cells carrying these constructs were individually cultured, resuspended with the infiltration buffer containing 10 mM MgCl_2_, 10 mM MES (pH5.8), and 100 μM acetosyringone, and infiltrated into 4-week-old wild-type *N. benthamiana* or RFP-H2B transgenic *N. benthamiana* leaves as described (Yang et al., 2018). For co-infiltration experiments, *A. tumefaciens* cultures harboring different constructs were adjusted to an optical density OD_600_ = 1.0 and mixed in equal proportion before infiltration.

### Confocal Microscopy

Imaging of fluorescent proteins in the epidermal cells of agroinfiltrated *N. benthamiana* or RFP-H2B plants was performed on a laser-scanning confocal microscope (LSM880; Carl Zeiss, Jena, Germany) at 36–48 hours post-infiltration (hpi). Leaf tissues were examined using a Zeiss c-Apochromat 40 × 1.2 water immersion Korr objective. The GFP fluorophore was excited with 488-nm laser lines and emission was detected at 500–550 nm. To avoid chlorophyll auto-fluorescence, the RFP fluorophore was excited with 561-nm laser lines and emission was detected at 600–630 nm. The YFP fluorophore was excited with 514-nm laser lines and emission was detected at 519–587 nm. The CFP fluorophore was excited with 458-nm laser lines and emission was detected at 470–500 nm. For every experimental sample, at least three independent biological replicates were examined. Images were processed with Zeiss ZEN software.

### Plant RNA Extraction and Analysis

Total RNA was extracted from infiltrated leaf regions of RFP-H2B plants using TRIzol reagent (Invitrogen, Carlsbad, CA, United States) following the manufacturer’s instructions. Complementary DNA (cDNA) was synthesized from 1 μg of total RNA using PrimeScript RT reagent Kit with genomic DNA Eraser (Takara, Dalian, China). Expression of the individual ORF of MMDaV was detected by reverse transcription PCR (RT-PCR) from the synthesized cDNA as previously described (Yang et al., 2018).

### Analysis of Protein Expression

Total plant proteins were extracted from infiltrated leave patches as described (Yang et al., 2011). Supernatant of extracted proteins was resolved by 12.5% SDS-PAGE, and transferred to nitrocellulose membranes which were then blocked and probed with primary antibodies raised against GFP (Roche Applied Science, Basel, Switzerland), HA (Santa Cruz, California, CA, United States), or FLAG (Sigma-Aldrich, St Louis, MO, United States). After washing, the membranes were incubated with a secondary peroxidase-conjugated goat antimouse antibody (Cell Signaling Technology, Boston, MA, United States). The results were visualized using a chemiluminescence detection system (Tianneng, Shanghai, China).

### Yeast Two-Hybrid Assays

GAL4-based yeast two-hybrid (Y2H) assays were carried out following the Clontech yeast protocol handbook. In brief, both plasmids containing fusion proteins with the GAL4 activation domain and DNA-binding domain were cotransformed into the yeast strain Y2H Gold and plated onto a selective medium lacking tryptophan and leucine (SD/-Trp/-Leu) at 30°C for 3–5 days to select for doubly transformed yeast cells. Transformants were then transferred to the selective medium lacking leucine, histidine, and tryptophan (SD/-Leu/-His/-Trp) containing 5 mM 3-amino-1,2,4-triazole (3-AT) or the selective medium lacking adenine, histidine, leucine and tryptophan (SD/-Ade/-His/-Leu/-Trp) and cultured at 30°C for 3–5 days to identify possible interaction by activation of reporter genes His3 and/or Trp.

## Results

### V2 Colocalizes With Fibrillarin in the Nucleolus

**FIGURE 1:**
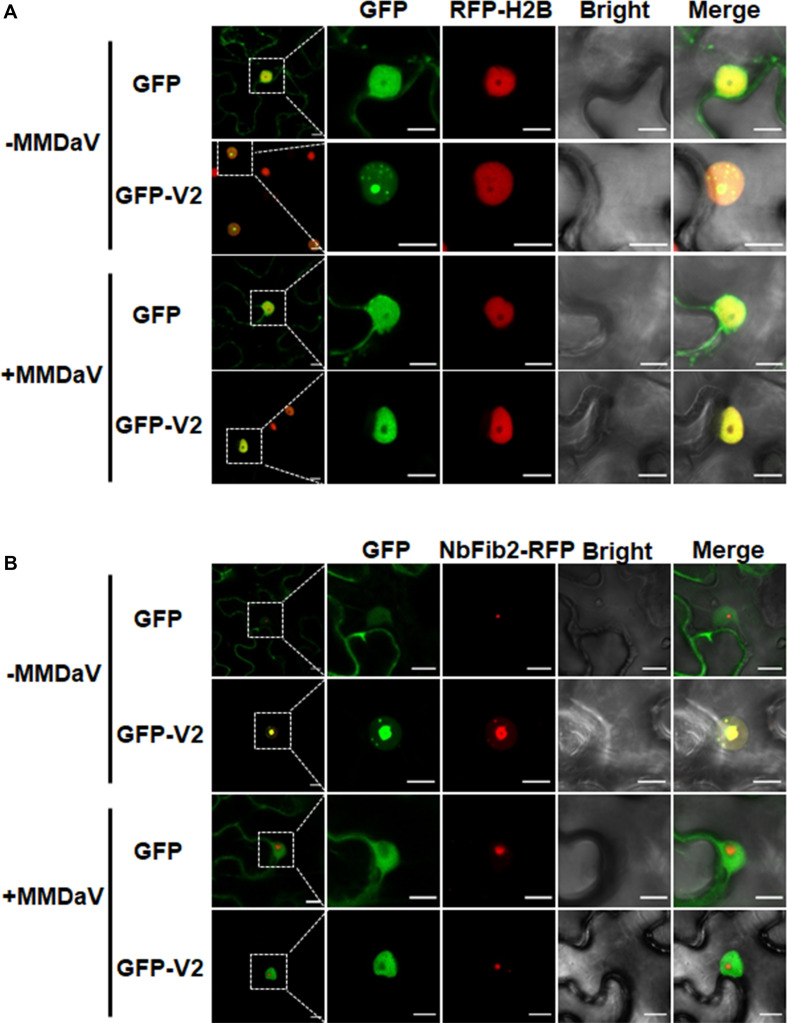
V2 localization within the nucleolus is modulated in the presence of mulberry mosaic dwarf-associated geminivirus (MMDaV). **(A)** Subcellular localization of green fluorescent protein (GFP) or GFP-V2 fusion in the absence or presence of MMDaV infection in transgenic *Nicotiana benthamian*a plants expressing red fluorescent protein (RFP)-tagged histone 2B (RFP-H2B). RFP-H2B was used as a nuclear marker. **(B)** Colocalization analysis of V2 and fibrillarin 2 (NbFib2) in the absence or presence of MMDaV in *N. benthamiana* plants. To create an environment mimicking MMDaV infection, the infectious clone of MMDaV was infiltrated into RFP-H2B or *N. benthamiana* leaves 12 h prior to the infiltration of GFP or GFP-V2. Images were taken using Zeiss LSM 880 confocal laser scanning microscope at 36 hours post infiltration (hpi) of GFP or GFP-V2. This experiment was done three times and more than 20 cells were observed per sample and replicate. A representative image is shown for each set. The corresponding region in the white box in column 1 is magnified and shown from Column 2 to Column 5. Scale bars correspond to 10 μm.

To better understand the subcellular localization of MMDaV V2, we took advantage of the transgenic *N. benthamiana* plants expressing RFP-H2B (hereafter designated as RFP-H2B plants). These plants contain a 2 × 35 Spro:RFP-H2B expression cassette which allow us to monitor the nucleoplasm but not the nucleolus of plant cells (Martin et al., 2009). We set out to transiently infiltrate the agrobacteria cultures harboring the GFP-V2 fusion construct (GFP was fused to the N-terminal of V2) into the leaves of RFP-H2B plants and visualized the fluorescence by confocal laser-scanning microscopy (CLSM) at 36 hpi. As a control, agrobacteria cultures containing construct solely expressing GFP was infiltrated into the leaves of RFP-H2B plants. Consistent with the previous study (Yang et al., 2018), V2 accumulates in the cytoplasm and nucleus (Supplementary Figure S1A). A close-up view of the nucleus showed that V2 accumulated to the subnuclear bodies of varying numbers (from approximately three to 12, with a mean number of about six), and these subnuclear bodies formed aggregates in about 25% of the observed cells (*n* = 60) (Supplementary Figure S1B). Despite of the varied number of subnuclear bodies in different cells, V2 was observed to target to a subnuclear dot resembling a localization within the nucleolus in all the detected cells (Figure 1A). This was in contrast to the GFP alone infiltrated RFP-H2B plant leaves, where GFP was excluded from the nucleolar regions (Figure 1A).

To further establish the ability of MMDaV V2 to localize in the nucleolus, we began to determine if the V2 protein colocalizes with fibrillarin, one of the major nucleoprotein that is considered as a typical marker for the subnuclear compartment (Zheng et al., 2016). As described previously (Zheng et al., 2016), confocal imaging of *N. benthamiana* plant leaves expressing NbFib2-RFP with GFP showed that NbFib2 localized to the nucleolus and Cajal body of the epidermal cells of *N. benthamiana* plant leaves (Figure 1B). Besides, GFP was excluded from the nucleolar regions in *N. benthamiana* leaves co-infiltrated with GFP and NbFib2-RFP (Figure 1B). However, *N. benthamiana* plant leaves expressing GFP-V2 and NbFib2-RFP and imaged by CLSM showed clear colocalization of V2 with NbFib2 mainly in the nucleolus (Figure 1B), indicating that V2 is present in the nucleolus of plant cells.

### V2 Interacts With NbFib2

**FIGURE 2:**
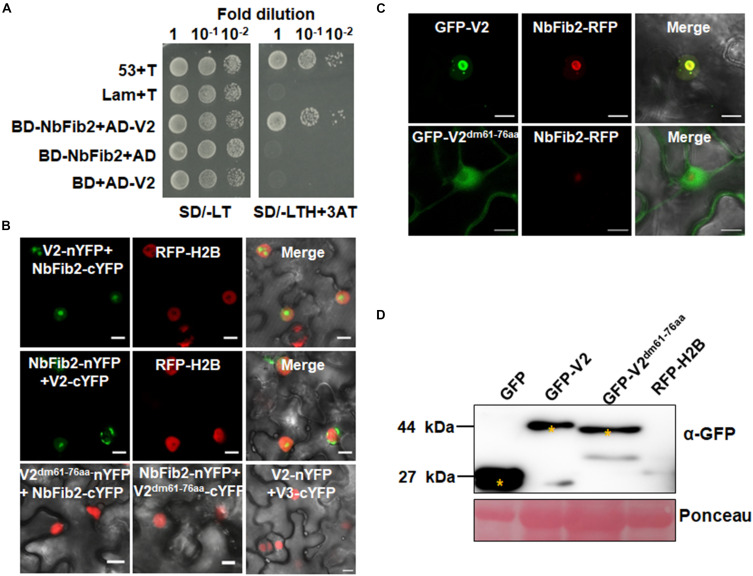
V2 interacts with NbFib2. **(A)** Yeast two-hybrid assay showing the interaction between V2 and NbFib2 in yeast cells. Full-length NbFib2 was expressed as GAL4 DNA-binding domain fusion (BD, bait) and V2 was expressed as GAL4 activation domain fusion (AD, prey) in yeast cells of the strain Y2H Gold. The interaction of p53 and T was used as a positive control, and cotransformation of Lam and T was used as a negative control. Growth on the plates lacking leucine and tryptophan (SD/-LT) indicates successful transformation of both prey and bait vectors, respectively. Interaction between NbFib2 and V2 is indicated by growth of yeast cells on media also lacking histidine supplementing with 5 mM 3-amino-1,2,4-triazole (SD/-LTH + 3-AT). **(B)** Bimolecular fluorescence complementation assay (BiFC) assay showing the interaction between NbFib2 and V2 in plant cells. Constructs containing N-terminal YFP fusion (nYFP) and C-terminal YFP fusion (cYFP) fusions were infiltrated into RFP-H2B plant leaves. Combinations of BiFC constructs are shown at the top of each panel. Images were taken using a Zeiss LSM 880 confocal laser scanning microscope at 48 hpi. Reconstituted YFP signals resulting from V2-NbFib2 interaction are displayed as a false-green color. RFP-H2B served as a nuclear marker. Note that deletion of the predicted nuclear localization signal (from amino acid 61–76) of V2 abolishes its interaction with NbFib2. **(C)** Colocalization analysis of NbFib2 with V2 and V2 mutant in the epidermal cells of *N. benthamiana* by Zeiss LSM 880 confocal laser scanning microscope at 36 hpi. At least 60 cells from three repeats were examined. Scale bars correspond to 10 μm. **(D)** Immunoblot of proteins from RFP-H2B plant leaves infiltrated with construct as indicated using anti-GFP antibody. Ponceau staining of the large subunit of Rubisco serves as a loading control.

As confocal microscopy suggests a colocalization of V2 and NbFib2 in the nucleolus of plant cells, a Y2H assay was performed to determine whether V2 directly interacts with NbFib2. V2 and NbFib2 was cloned into both of the yeast two-hybrid “prey” (Y2H) pGADT7 and “bait” pGBKT7 vectors to produce AD and BD fusions, and the bait and prey plasmids were then cotransformed to yeast strain Y2H Gold. Cotransformation of murine tumor suppressor p53 and simian virus 40 large T antigen (T) was used as a positive control and that of human lamin (Lam) and T as a negative control. Similar to the positive control, robust growth of yeast transformants containing BD-NbFib2 and AD-V2 was observed on the selective medium lacking histidine, leucine, and tryptophan supplementing with 5 mM 3-AT, whereas yeast transformants harboring AD-V2 with the empty BD vector or BD-NbFib2 with the empty AD vector failed to grow on the selective triple dropout medium, suggesting the specific interaction between NbFib2 and V2 in yeast cells (Figure 2A). NbFib2-V2 interaction was then confirmed in plant cells using BiFC. While no signal was detected for the negative control combination of V2–V3, YFP signal was evident in the cells co-infiltrated with the BiFC combinations of V2-NbFib2 (Figure 2B). As shown in Figure 2B, coexpression of V2 fused to the N-terminal of YFP (V2-nYFP) and NbFib2 fused to the C-terminal of YFP (NbFib2-cYFP) or NbFib2-nYFP and V2-cYFP reconstituted YFP fluorescence primarily in the nucleolus and weakly in the nucleoplasm in about 67% of the YFP-detectable cells (*n* = 60), and occasionally in some structures around/outside the nucleus in about 33% of the cells (Figure 2B), indicating that V2 interacts with NbFib2.

Since mutation of the predicted nuclear localization signal (NLS, from amino acids 61–77) of MMDaV V2 abolished its subnuclear foci localization and PTGS suppression activity (Yang et al., 2018), we performed a BiFC assay to evaluate whether this MMDaV V2 mutant interacts with NbFib2. As shown in Figure 2B, the NLS mutant of V2 did not interact with NbFib2 (Figure 2B). We then detected whether the NLS mutant is able to colocalize with NbFib2. In contrast to the wild-type V2 protein, the NLS mutant of V2 localized to the nucleoplasm and cytoplasm, while NbFib2 localized to the nucleolus (Figure 2C). Immunoblot analysis of total protein from infiltrated leave samples showed that the NLS mutant does not affect the stability of the V2 protein (Figure 2D and Supplementary Figure S2). These results suggest that V2, but not the V2 NLS mutant, colocalizes and interacts with NbFib2.

### V2 Nucleolar Accumulation Is Modulated in the Context of Virus Infection

To assess whether the subcellular localization of V2 varies during virus infection, the infectious clone of MMDaV was infiltrated into RFP-H2B plant leaves 12 h prior to the infiltration of agrobacteria cultures of GFP or GFP-V2 to create a cellular environment mimicking a natural viral infection. As shown in Figure 1A, MMDaV infection modulated the accumulation of V2 in the nucleus. In particular, the distribution of GFP-V2 displayed relocalization throughout the nucleoplasm but not to the nucleolus in all detected cells (*n* = 60). However, coexpression of GFP with MMDaV did not cause any profound shift in the localization of GFP (Figure 1A). Western blot analysis of total protein from infiltrated leave patches with the antibody against GFP showed that both GFP and GFP-V2 were expressed in the absence or presence of MMDaV (Supplementary Figure S3). Since V2 colocalizes with NbFib2 in the nucleolus in the absence of MMDaV infection, we next investigated whether V2 and NbFib2 could still colocalize in the nucleolus in the presence of virus infection. To this end, infectious clone of MMDaV was infiltrated into *N. benthamiana* leaves 12 h before the coinfiltration of GFP-V2 with NbFib2-RFP. Using confocal microscopy, we observed that V2 could not colocalize with NbFib2 in the nucleolus in the context of MMDaV infection, i.e., NbFib2 accumulated mainly in the nucleolus, whereas V2 was found in the nucleoplasm (Figure 1B), suggesting that the capability of V2 localizing to the nucleolus changes in the context of MMDaV infection.

### RepA Interacts With V2 and Excludes V2 From the Nucleolus

**FIGURE 3:**
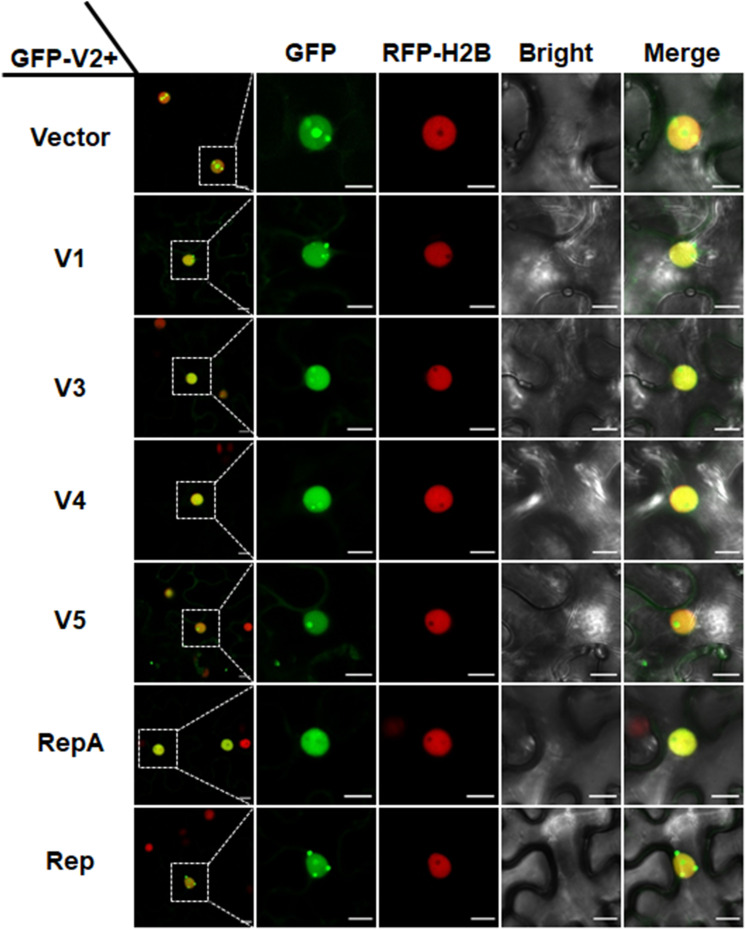
RepA-dependent nucleolar exclusion of V2. RFP-H2B plant leaves were infiltrated with *Agrobacterium tumefaciens* cultures carrying constructs to express GFP-V2 and the other six individual ORFs (V1, V3, V4, V5, RepA, and Rep) of MMDaV. Fluorescence was visualized under a Zeiss LSM 880 confocal laser scanning microscope at 36 hpi. The corresponding region in the white box in column 1 is magnified and shown from Column 2 to Column 5. RFP-H2B was used as a nuclear marker. This experiment was done three times and more than 20 cells were observed per sample and replicate. Scale bars correspond to 10 μm.

In animal viruses and TYLCV, virus-regulated dynamic localization of CPs is contributed to another viral protein(s) (Wistuba et al., 1997; Shishido-Hara et al., 2000). For example, the Rep protein of TYLCV can mediate the nucleolar exclusion of CP (Wang et al., 2017). Unlike all the identified monopartite geminiviruses, the distinct monopartite geminivirus MMDaV encodes five ORFs (V1, V2, V3, V4, and V5) and two ORFs (C1 or RepA, and C2 or Rep) on the virion-sense and the complementary-sense strands, respectively (Ma et al., 2015). To test whether any of another viral protein(s) of MMDaV is responsible for the differential localization of V2 associated with MMDaV infection, agrobacteria cultures harboring the construct of GFP-V2 was transiently infiltrated into RFP-H2B plant leaves together with bacterium cultures containing pCHF3 plasmids expressing each of the viral proteins including V1, V3, V4, V5, RepA, and Rep independently. Similar to what was seen in GFP-V2 alone infiltrated RFP-H2B plants, co-infiltration of GFP-V2 with any of the five proteins (V1, V3, V4, V5, and Rep) was not able to recapitulate the MMDaV-induced nucleolar exclusion of V2. Strikingly, when GFP-V2 was coinfiltrated with RepA, it no longer showed a nucleolar accumulation and instead was more evenly distributed throughout the nucleoplasm in all detected 60 cells (Figure 3). RT-PCR amplification of the individual MMDaV ORFs from the leaf disks used for confocal microscopy confirmed the expression of viral genes from the binary vectors (Supplementary Figure S4), indicating that RepA is sufficient to exclude V2 from the nucleolus.

**FIGURE 4:**
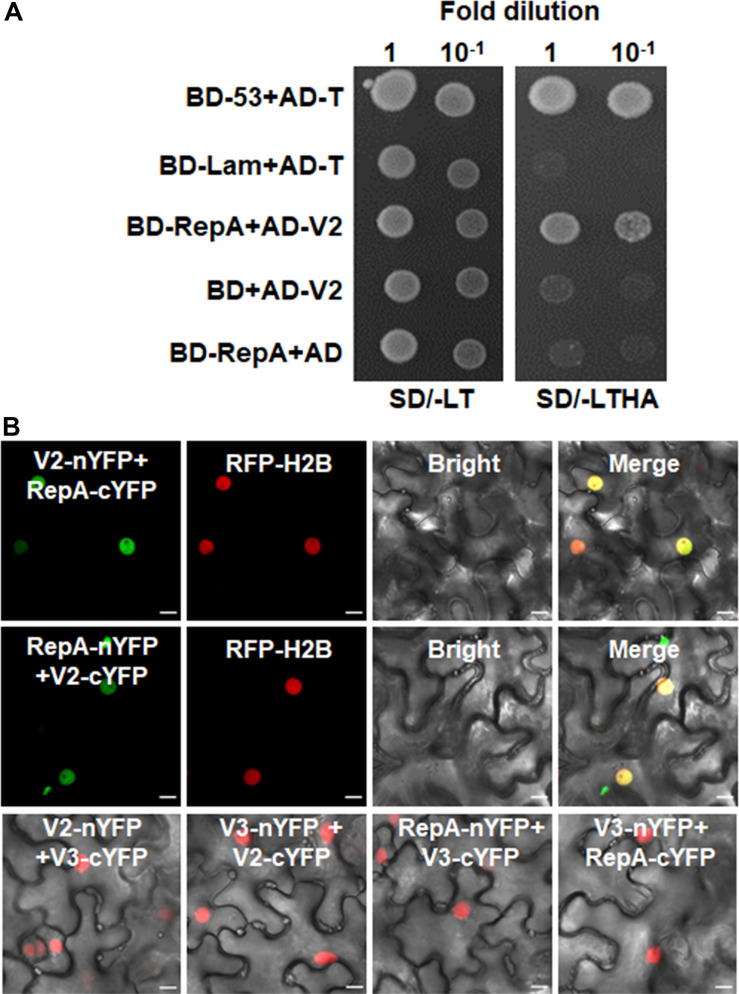
RepA interacts with V2 in yeast and plant cells. **(A)** Yeast two-hybrid assay showing the interaction between RepA and V2 in yeast cells. Growth of yeast cotransformants containing the BD-RepA and AD-V2 fusions on the plates lacking leucine, tryptophan, histidine, and adenine (SD/-LTHA) indicates specific interaction between RepA and V2. **(B)** BiFC assay showing the interaction between RepA and V2 in plant cells. Combinations of BiFC constructs are shown at the top of each panel. Images were taken using a Zeiss LSM 880 confocal laser scanning microscope at 48 hpi. Reconstituted YFP signals as a consequence of V2-RepA interaction are depicted as a false-green color. RFP-H2B served as a nuclear marker.

As RepA is sufficient to exclude V2 from the nucleolus, it raised the possibility that there might be a direct interaction between RepA and V2. To test the hypothesis, we explored both the Y2H and BiFC assay to examine the interaction of V2 and RepA. While the fusion protein BD-RepA interacted with the AD-V2 fusion, no activation of the reporter gene was observed when BD-RepA with AD or BD and AD-V2 was expressed in the yeast cells (Figure 4A). Further examination of the potential RepA-V2 interaction by the BiFC assay showed that the fluorescence due to the reconstitution of YFP *in planta* was only observed in the nucleoplasm by the BiFC combination of RepA and V2 (Figure 4B). However, no YFP signal was visible in any combination of the negative controls V2-nYFP + V3-cYFP, V3-nYFP + V2-cYFP, RepA-nYFP + V3-cYFP, and V3-nYFP + RepA-cYFP (Figure 4B), suggesting that V2 physically interacts with RepA. Collectively, our results reveal that RepA interacts with V2 and redistributes V2 from the nucleolus to nucleoplasm.

### RepA Excludes the Site of V2–V2 Interactions From the Nucleolus

**FIGURE 5:**
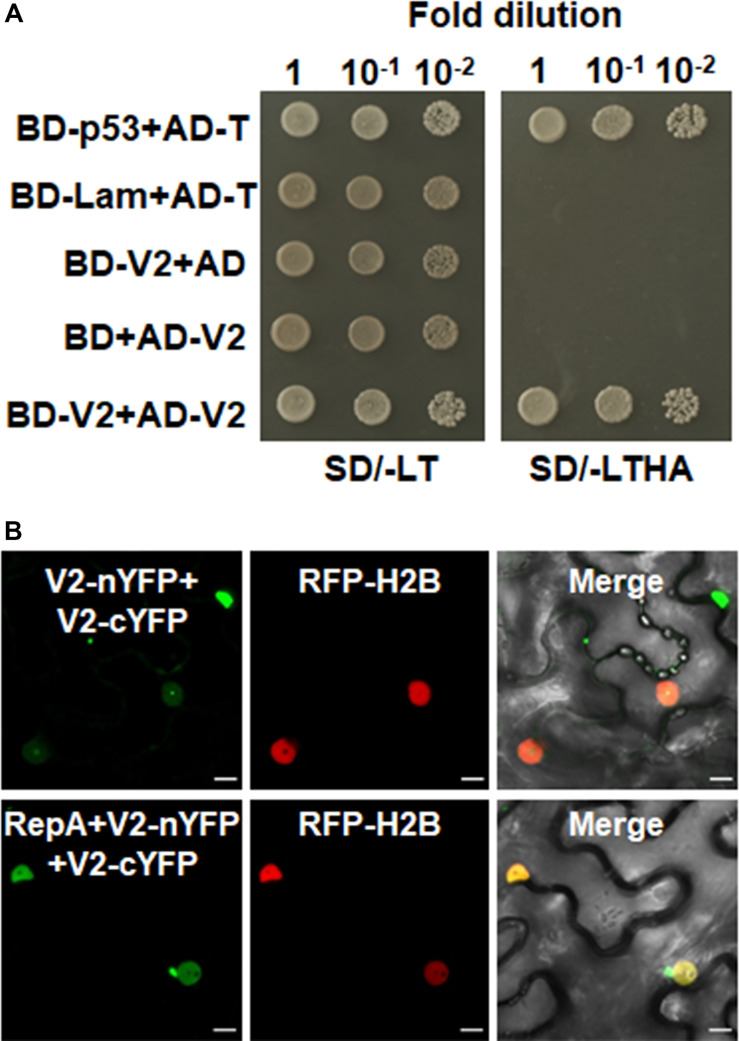
RepA changes the site of V2–V2 self-interaction. **(A)** Self-interaction of V2 determined by yeast two-hybrid assay. **(B)** BiFC assays in leave cells of RFP-H2B plants. Self-interaction of V2 in the absence or presence of RepA was examined at 48 hpi using a confocal microscope. YFP signal resulting from interacting protein combinations are indicated as green. RFP-H2B was a marker for the nucleus. Note that the sites of V2–V2 self-interaction was excluded from the nucleolus by RepA. Images are representative of three independent experiments, in each of which at least 20 cells were examined. Scale bars correspond to 10 μm.

Self-interaction of viral proteins play critical roles in many steps of the virus infection cycle. To determine whether V2 interacts with itself and whether RepA has any impact on V2 self-interaction, we first tested the self-interaction of V2 in yeast cells. Growth of yeast transformants harboring AD-V2 and BD-V2 in selective yeast medium plates lacking adenine, histidine, leucine, and tryptophan indicated the self-interaction of V2 in yeast cells (Figure 5A). The capacity for self-interaction of V2 was further determined by the BiFC assay. Coexpression of V2 fused to the N-terminal of YFP (V2-nYFP) and V2 fused to the C-terminal of YFP (V2-cYFP) reconstituted YFP fluorescence mainly in the nucleolus and weakly in the nucleoplasm (Figure 5B). However, when V2-nYFP and V2-cYFP was coexpressed with RepA, the reconstituted YFP fluorescence was reduced in the nucleolus and redistributed to the nucleoplasm in all the YFP-detectable cells (*n* = 60) (Figure 5B), suggesting that RepA also has a profound influence on the sites of V2–V2 interaction.

### RepA Changes the Interaction Site of V2 and NbFib2

**FIGURE 6:**
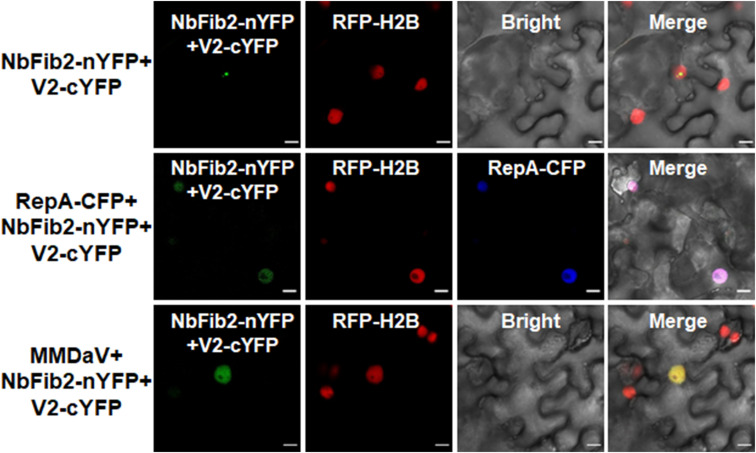
Representative images showing the impact of RepA and MMDaV on the localization of V2-NbFib2 complex. Agrobacteria cultures harboring a cyan fluorescent protein-tagged RepA (RepA-CFP) or infectious clone of MMDaV was coexpressed with the BiFC combination constructs of NbFib2-V2. Images were captured at 48 hpi. Scale bars correspond to 10 μm.

As V2 colocalized and interacted with NbFib2 in the nucleolus, we tried to determine whether RepA mediates nucleolar exclusion of V2 by impacting the V2-NbFib2 interaction. For this purpose, a CFP-tagged RepA (RepA-CFP) construct was generated and coexpressed with the BiFC combination of V2 and NbFib2. Interestingly, when RepA-CFP was coexpressed with NbFib2-nYFP and V2-cYFP in RFP-H2B plant leaves, an altered pattern of BiFC signal distribution was detected in infiltrated cells. Coexpression of the RepA-CFP protein with NbFib2-nYFP and V2-cYFP consequently redistributed the V2-NbFib2 complex out of the nucleolus to the nucleoplasm (Figure 6). Exclusion of the V2-NbFib2 complex from the nucleolus was also detected upon the infiltration of *N. benthamiana* plant leaves with MMDaV, NbFib2-nYFP, and V2-cYFP (Figure 6). These data shows that RepA is able to redirect the V2-NbFib2 complexes to the nucleoplasm during MMDaV infection.

## Discussion

Geminiviruses are known to have a limited coding capacity and have compensated for this restriction by encoding multifunctional viral proteins to make use of host biosynthetic machinery and to suppress multiple layers of plant immune responses to achieve a successful infection (Shunmugiah et al., 2017; Yang et al., 2019). Perhaps as a consequence, geminivirus multifunctional proteins could be distributed in various compartments of a plant cell to establish contacts with different host and viral components to fulfill their multiple tasks. In this study, we compared the subcellular localization of MMDaV V2 in non-infected and infected plant cells. We observed the modulated subcellular distribution of V2 in response to MMDaV infection. While the V2 protein accumulates in the nucleolus upon transient expression in the epidermal cells of *N. benthamiana* plants, V2 is excluded from the nucleolus in cells infected with MMDaV. This localization difference of V2 is associated to the presence of the RepA protein of MMDaV.

Viral proteins targeting the nucleus generally rely on a nuclear localization signal consisting of a short stretch of basic residues that allows interaction with the nuclear import proteins, importins, mediating entry into the nucleus via the nuclear pore complex (Lange et al., 2007). In contrast, despite many different sequences that are able to direct plant or viral proteins to the nucleolus have been identified, there is no consensus signal for nucleolar localization. The localization of a viral protein to the nucleolus largely attributes to direct or indirect interaction with other nucleolar proteins (Kalinina et al., 2018). We have shown here that the MMDaV V2 protein colocalizes with NbFib2 in the nucleolus of plant cells. The specific interaction between V2 and NbFib2 was confirmed by both the yeast two-hybrid and BiFC assays. Fib2 is a key component of several ribonucleoproteins that has been implicated to interact with an increasing number of viral proteins. The outcomes of viral protein and Fib interactions vary depending on different viruses. On the one hand, viral proteins, such as BSMV TGB1, GRV ORF3, and the P20 protein of BaMV satRNA, hijack Fib2 for virus cell-to-cell or systemic movement (Kim et al., 2007; Chang et al., 2016; Li et al., 2018). One the other hand, viral proteins may associate with Fib to counter RNA silencing-based host defense (Rajamäki and Valkonen, 2009). Previously, it was shown that deletion of the basic motif encompassing amino acids 61 to 76 abolished the subnuclear localization and PTGS suppression activity of MMDaV V2 (Yang et al., 2018). In the present study, we showed that this MMDaV V2 mutant could neither interact nor colocalize with NbFib2. It would be interesting to determine whether V2-NbFib2 interaction is required for V2 to suppress RNA silencing.

Besides its accumulation in the nucleolus, MMDaV V2 also targets to other subnuclear foci outside of the context of virus infection. Note that the numbers of the subnuclear foci are approximations as it is difficult to assess due to their unclear boundaries and aggregation in the nucleus in some cases (Supplementary Figure S1B). Interestingly, targeting to subnuclear bodies have also been observed for GRBaV V3, though what the GRBaV-containing subnuclear bodies correspond to needs further study (Guo et al., 2015). It is also interesting to note that TYLCV CP can be detected in numerous nuclear foci in the context of virus infection. This virus-induced CP-targeting nuclear speckles can partially colocalize with markers of sites of RNA processing, raising the possibility of CP in interference with RNA metabolism (Wang et al., 2017). Further identification of the subnuclear bodies at which MMDaV V2 localizes would give a better understanding of the subcellular localization and potential biological role of MMDaV V2.

Strikingly, V2 is excluded from the nucleolus to the nucleoplasm in plant cells infected with MMDaV. Although this virus-dependent exclusion of V2 from the nucleolus might reflect more the natural situation in the viral infection process, we cannot rule out the possibility of temporal and spatial distribution of V2 to the nucleolus. Transporting of viral protein to different subcellular compartments has also been implicated to play critical roles in functional specification of plant viruses. For example, the nuclear-cytoplasmic compartmentalization of cucumber mosaic virus (CMV) 2b presumably maximizes the benefit for CMV by regulating the balance between virus accumulation and damage to the host (Du et al., 2014). Nucleocytoplasmic shuttling of tomato leaf curl Yunnan Virus (TLCYnV) C4 is critical for its function as a pathogenicity factor (Mei et al., 2018). Localization of the TYLCV V2 protein to the cajal body is required for V2 to interact with AGO4 and to suppress DNA methylation-mediated TGS against TYLCV (Ding and Lozano-Duran, 2020; Wang et al., 2020). Although very little is understood about the biological significance of MMDaV V2 translocation in virus infection, it raises a new perspective that the exclusion of V2 to the nucleoplasm might represent a new role for V2.

We also presented evidence that the dynamic localization of V2 and V2–V2 self-interaction site is RepA-dependent. We found that RepA redirects the V2-NbFib2 complex from the nucleolus to the nucleoplasmic sites. As RepA is involved in geminivirus replication, RepA-V2 interaction-mediated nucleolar exclusion of V2 might imply a link between virus replication and silencing suppression. Recruitment of the BSMV γb protein by the αa viral replication protein to the chloroplast membrane site of BSMV replication enhances the helicase activities of αa in unwinding of dsRNA duplexes and promotes BSMV replication (Zhang et al., 2017). It needs to be determined whether the nuclear export of V2 has any effect on RepA function or virus replication.

## Conclusion

We have shown that MMDaV V2 displays a differential subcellular localization pattern in response to MMDaV infection. We also show that RepA interacts with V2 and mediates the nucleolar exclusion of V2. As many different viruses target the nucleolus to aid virus infection, the study of the interaction of viral proteins with the nucleolus will provide insight into the mechanisms of viral manipulating host cell functions which could lead to the design of novel antiviral strategies.

## Data Availability Statement

All datasets generated for this study are included in the article/Supplementary Material.

## Author Contributions

DW, SS, YR, and XY performed the experiments. All the authors contributed to the experimental design and interpretation. XY, DW, and XZ wrote the manuscript with contributions from all authors.

## Conflict of Interest

The authors declare that the research was conducted in the absence of any commercial or financial relationships that could be construed as a potential conflict of interest.
