# Supplementary material for: RepA Promotes the Nucleolar Exclusion of the V2 Protein of Mulberry Mosaic Dwarf-Associated Virus
Source: Front Microbiol. 2020 Aug 4;11:1828. doi: 10.3389/fmicb.2020.01828 (PMC7438950; doi:10.3389/fmicb.2020.01828)
Supplement: Supplementary file 1 [file Table_1.docx]

**Table S1 Synthetic oligonucleotide primers used in this study**

| Primers | Primer sequences (5’-3’) |
| --- | --- |
| V2-GW-F | CACCatgtctttgtggagtaccaaattag |
| V2-GW-R | attccaaatgtgccacgtttc |
| NbFib2-GW-F | CACCATGGTTGCACCAACTAGAGGTCGCG |
| NbFib2-GW-R | ggcagcagccttctgcttcttc |
| RepA-GW-F | CACCatggcttcaagttctaacttcag |
| RepA-GW-R | aagatctggcccattgc |
| V3-GW-F | CACCatgagctataaatacccccctgc |
| V3-GW-R | cggcactgagtaaggtg |
| V2-EcoRI-F | GAATTCatgtctttgtggagtaccaaattag |
| V2-BamHI-R | attccaaatgtgccacgtttc |
| NbFib2-EcoRI-F | GAATTCATGGTTGCACCAACTAGAGGTCGCG |
| NbFib2-BamHI-R | GGATCCggcagcagccttctgcttcttc |
| RepA-EcoRI-F | GAATTCatggcttcaagttctaacttcag |
| RepA-BamHI-R | GGATCCctaaagatctggcccattgc |
