# Supplementary material for: RepA Promotes the Nucleolar Exclusion of the V2 Protein of Mulberry Mosaic Dwarf-Associated Virus
Source: Front Microbiol. 2020 Aug 4;11:1828. doi: 10.3389/fmicb.2020.01828 (PMC7438950; doi:10.3389/fmicb.2020.01828)
Supplement: Supplementary file 2 [file Presentation_1.pdf]

FIGURE S1. Confocal microscopy of GFP-V2 at 36 hours post infiltration (hpi). RFP-H2B plant leaves were infiltrated with *Agrobacterium tumefaciens* harboring a construct to express GFP-V2. **(A)** Images were captured with a higher gain setting to better visualize the localization of V2 to the cytoplasm. **(B)** Images were captured with a lower gain setting for GFP to better visualize the localization of V2 to the subnuclear bodies. RFP-H2B was used as a nuclear marker. Bars represent 10 μm.

FIGURE S2. Immunoblot of half-YFP fusion proteins in agroinfiltrated leaves at two days post infiltration (dpi). The nYFP and cYFP fusion constructs are shown at the top of each panel as indicated. The molecular weight of each target protein is shown on the left and the primary antibodies used for the immunoblot are indicated on the right of each panel. Yellow stars indicate the target protein. Ponceau staining of the large subunit of Rubisco serves as a loading control.

FIGURE S3. Detection of the expression of GFP and GFP-V2 in the absence or presence of MMDaV infection using anti-GFP antibody. RFP-H2B plant leaves were infiltrated with *A. tumefaciens* harboring the constructs as designated. Proteins were extracted from plant samples at 36 hpi. The molecular weight of each target protein is shown on the left. Yellow stars indicate the target protein. Ponceau staining of the large subunit of Rubisco serves as a loading control.

FIGURE S4. Reverse transcript PCR (RT-PCR) analysis of the expression of viral genes in the samples of Figure 3. Samples were collected for RNA extraction and subsequent cDNA synthesis after confocal microscopy. *A. tumefaciens* containing the independent viral gene was used as a positive control (+) for PCR amplification. cDNA of samples infiltrated with empty pCHF3 vector and GFP-V2 (Mock) was used as a negative control to show the specificity of RT-PCR. The molecular ladder is shown on the left. Target viral genes are indicated on the top of each panel.

FIGURE S1

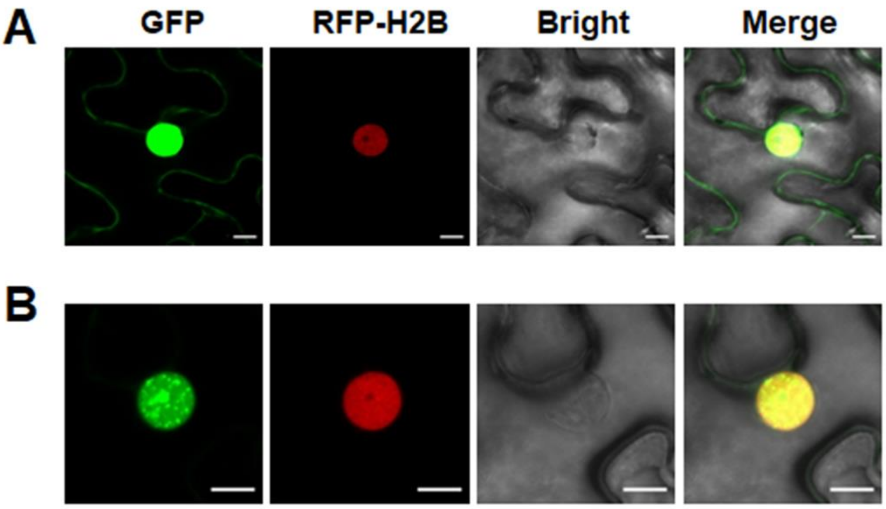

FIGURE S2

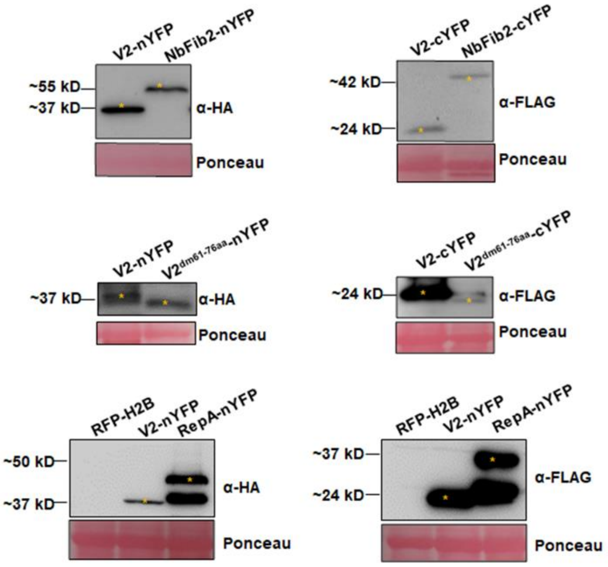
V2-nYFP
NbFib2-nYFP
~55 kD
~37 kD
α-HA
Ponceau
V2-cYFP
NbFib2-cYFP
~42 kD
~24 kD
α-FLAG
Ponceau
V2-nYFP
V2dm61-76aa-nYFP
~37 kD
α-HA
Ponceau
V2-cYFP
V2dm61-76aa-cYFP
~24 kD
α-FLAG
Ponceau
RFP-H2B
V2-nYFP
RepA-nYFP
~50 kD
~37 kD
α-HA
Ponceau
RFP-H2B
V2-nYFP
RepA-nYFP
~37 kD
~24 kD
α-FLAG
Ponceau

FIGURE S3

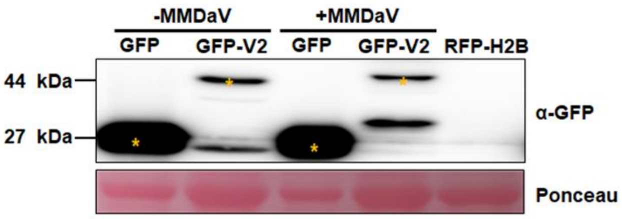

-MMDaV
+MMDaV
GFP
GFP-V2
GFP
GFP-V2
RFP-H2B
44 kDa
27 kDa
α-GFP
Ponceau

FIGURE S4

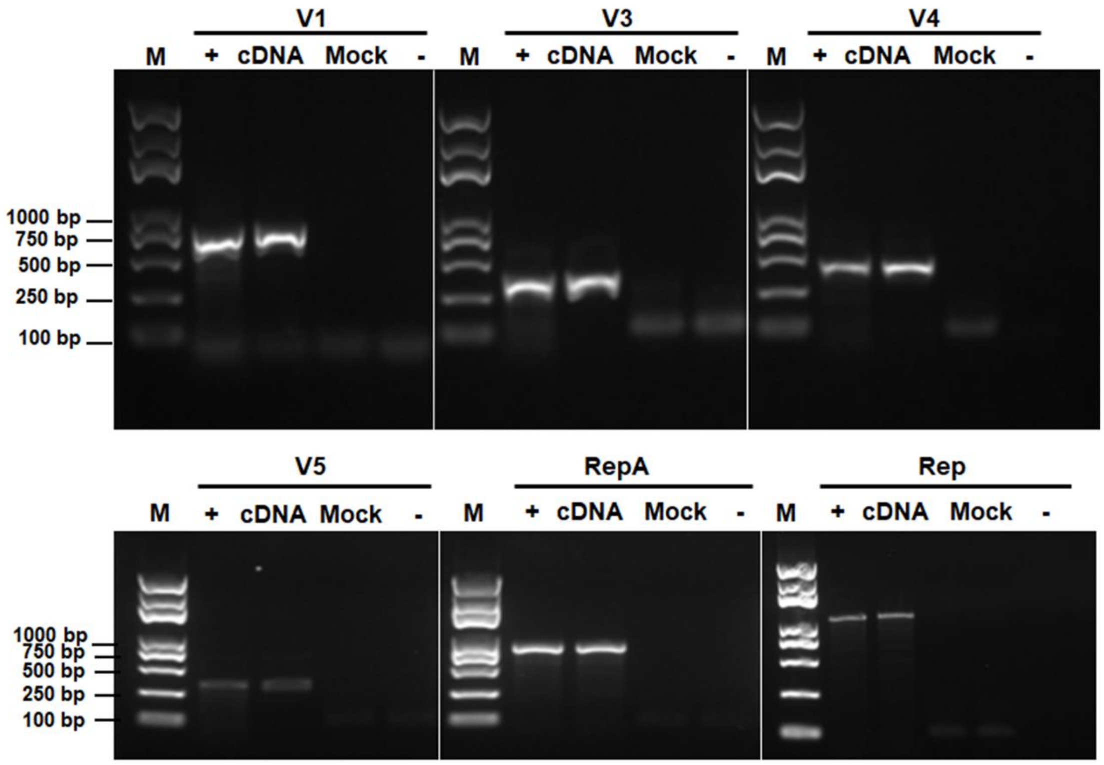

V1
V3
V4
M
+
cDNA
Mock
-
1000 bp
750 bp
500 bp
250 bp
100 bp
V5
RepA
Rep
